# YAP, TAZ, and Hippo-Dysregulating Fusion Proteins in Cancer

**DOI:** 10.1146/annurev-cancerbio-061223-094639

**Published:** 2024-06

**Authors:** Jordan H. Driskill, Josephine K. Dermawan, Cristina R. Antonescu, Duojia Pan

**Affiliations:** 1 Department of Physiology, Howard Hughes Medical Institute, University of Texas Southwestern Medical Center, Dallas, Texas, USA; 2 Department of Pathology and Laboratory Medicine, Weill Cornell Medicine, New York, NY, USA; 3 Department of Pathology, Memorial Sloan Kettering Cancer Center, New York, NY, USA; 4 Robert J. Tomsich Pathology and Laboratory Medicine Institute, Cleveland Clinic, Cleveland, Ohio, USA

**Keywords:** YAP1, WWTR1/TAZ, Hippo pathway, gene fusions, sarcoma, cancer

## Abstract

Gene fusions are well-known drivers of cancer and are potent targets for molecular therapy. An emerging spectrum of human tumors harbors recurrent and pathognomonic gene fusions that involve the transcriptional coactivator *YAP1* (which encodes the protein YAP) or its paralog *WWTR1* (which encodes the protein TAZ). YAP and TAZ are frequently activated in cancer and are the transcriptional effectors of the Hippo pathway, a highly conserved kinase cascade that regulates diverse functions such as organ size, development, and homeostasis. In this review, we discuss the tumors that have YAP, TAZ, or other Hippo-dysregulating fusion proteins; the mechanisms of these fusion proteins in driving their respective tumors; and the potential vulnerabilities of these chimeric oncoproteins across cancers of many origins. Furthermore, as new *YAP1* and *WWTR1* gene fusions are discovered, we provide a framework to predict whether the resulting protein product is likely to be oncogenic.

## INTRODUCTION

Since the discovery of the highly recurrent Philadelphia chromosome in chronic myelogenous leukemia (CML) (Nowell 1962), structural chromosomal rearrangements in cancer have been well appreciated to result in pathogenic gene fusions (Mitelman et al. 2007). As next-generation sequencing and unbiased fusion gene detection software become more prevalent in clinical discovery, an increasing number of gene fusions are being reported in neoplasia, though the function of many of these gene fusions remains unknown (Mertens et al. 2015). However, the success of targeted molecular therapy, such as imatinib toward the *BCR::ABL* gene fusion in CML (Druker 2008; Druker et al. 2001a,b), larotrectinib toward NTRK-fusion-positive cancers (Drilon et al. 2018), and ALK inhibitors toward ALK-fusion-positive cancers (Lin et al. 2017), provides hope that targeting additional gene fusions may inhibit a variety of cancers for which there are few therapeutic options.

**Figure 1:**
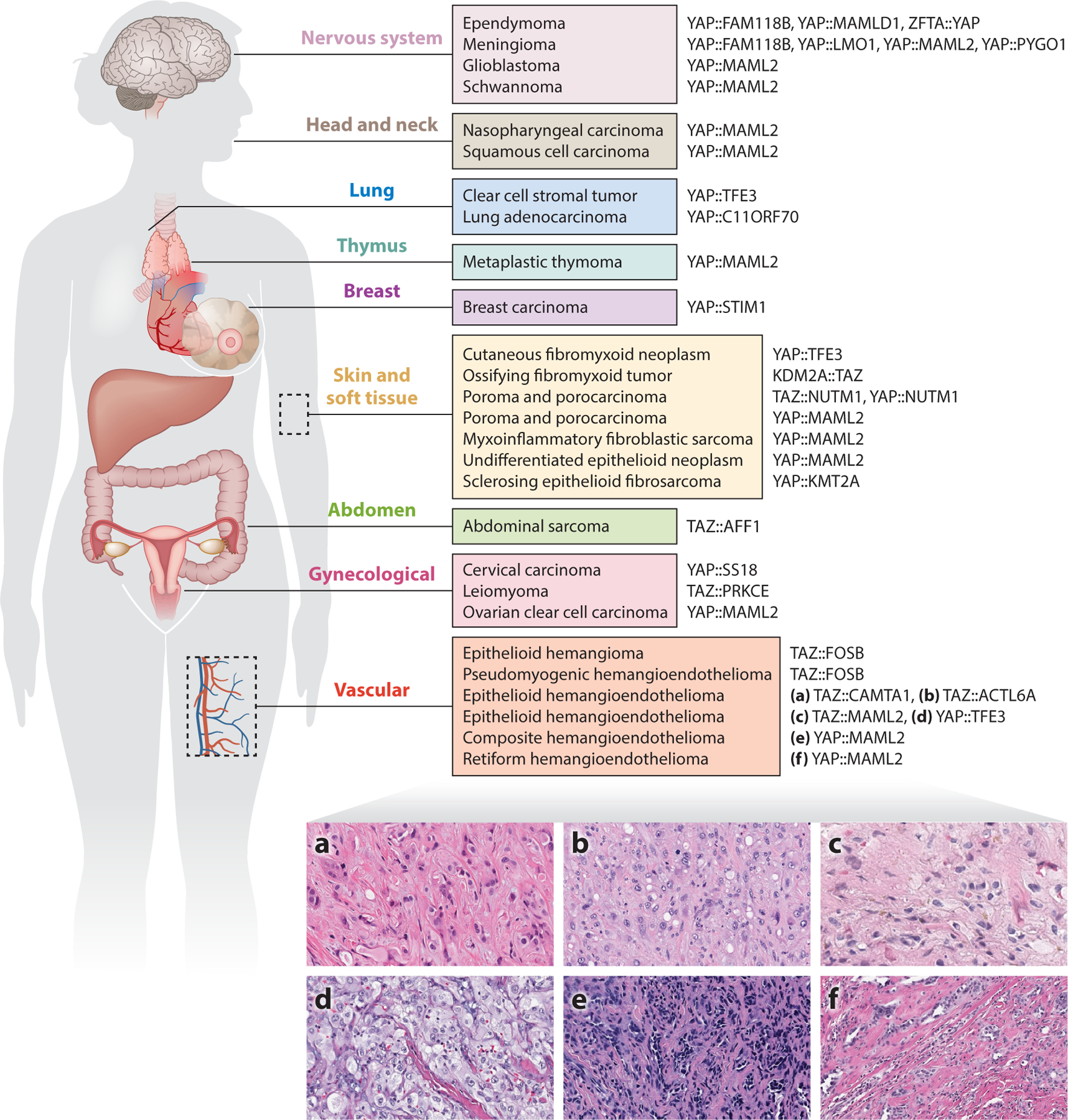
Tumors with reported YAP/TAZ fusion proteins. Tumor names are shown adjacent to their corresponding YAP/TAZ fusion protein(s). Panels *a*–*f* show the variability of histology of YAP-/TAZ-fusion-associated hemangioendotheliomas. (*a*) TAZ::CAMTA1 epithelioid hemangioendothelioma (EHE) showing epithelioid tumor cells with pale eosinophilic cytoplasm, occasional intracytoplasmic vacuoles, open chromatin, and inconspicuous nucleoli arranged in clusters and cords against background myxohyaline stroma. (*b*) TAZ::ACTL6A EHE showing epithelioid tumor cells with granular eosinophilic cytoplasm, frequent intracytoplasmic vacuoles, vesicular chromatin, and variably prominent nucleoli arranged in small nests and solid sheets set in a collagenous to fibrous stroma. (*c*) TAZ::MAML2 EHE showing epithelioid cells with pale eosinophilic cytoplasm, occasional intracytoplasmic vacuoles, and open chromatin arranged haphazardly against background myxohyaline stroma and admixed with hemosiderin and occasional erythrocytes. (*d*) YAP::TFE3 EHE displaying large epithelioid cells with voluminous glassy cytoplasm and variably prominent nucleoli arranged in nests and solid sheets. (*e*) YAP::MAML2 composite hemangioendothelioma containing an admixture of arborizing vascular channels and solid areas of uniform hyperchromatic ovoid to cuboidal tumor cells. (*f*) YAP::MAML2 retiform hemangioendothelioma consisting of arborizing and elongated vascular channels lined by hobnail endothelial cells dissecting through dermal connective tissue.

Recent discoveries have unveiled previously unknown gene fusions within the vascular malignancy epithelioid hemangioendothelioma (EHE): the common *WWTR1::CAMTA1* gene fusion and the rare *YAP1::TFE3* gene fusion (Antonescu et al. 2013, Errani et al. 2011, Tanas et al. 2011). Moreover, variant *WWTR1* and *YAP1* gene fusions have been identified in cardiac EHE (Suurmeijer et al. 2020) and subsets of retiform and composite hemangioendotheliomas (Antonescu et al. 2020), respectively (Figure 1). As TAZ (the common name of the protein encoded by *WWTR1* and not to be confused with Tafazzin whose gene name is *TAZ*) and its paralog YAP (encoded by *YAP1*) are the transcriptional effectors of the Hippo pathway (Zheng & Pan 2019), the discovery of these gene fusions in EHE suggested a new potential role for YAP and TAZ fusion proteins in cancer. YAP and TAZ fusion proteins would soon be found to define subtypes of supratentorial ependymoma (Pajtler et al. 2015, Parker et al. 2014), poroma and porocarcinoma (Sekine et al. 2019), and an increasing number of solid tumors (Garcia et al. 2022, Szulzewsky et al. 2021).

In this review, we discuss the discoveries of these YAP and TAZ fusion proteins in a variety of different tumors, their known pathogenic mechanisms, and their molecular vulnerabilities that can potentially be exploited for therapy. We also discuss an increasing understanding of how other known fusion genes dysregulate Hippo signaling to activate YAP and TAZ. Furthermore, we provide a new framework that will allow researchers to better understand YAP and TAZ fusion genes as they become discovered in novel rearrangements in new tumor types.

## THE HIPPO PATHWAY

**Figure 2:**
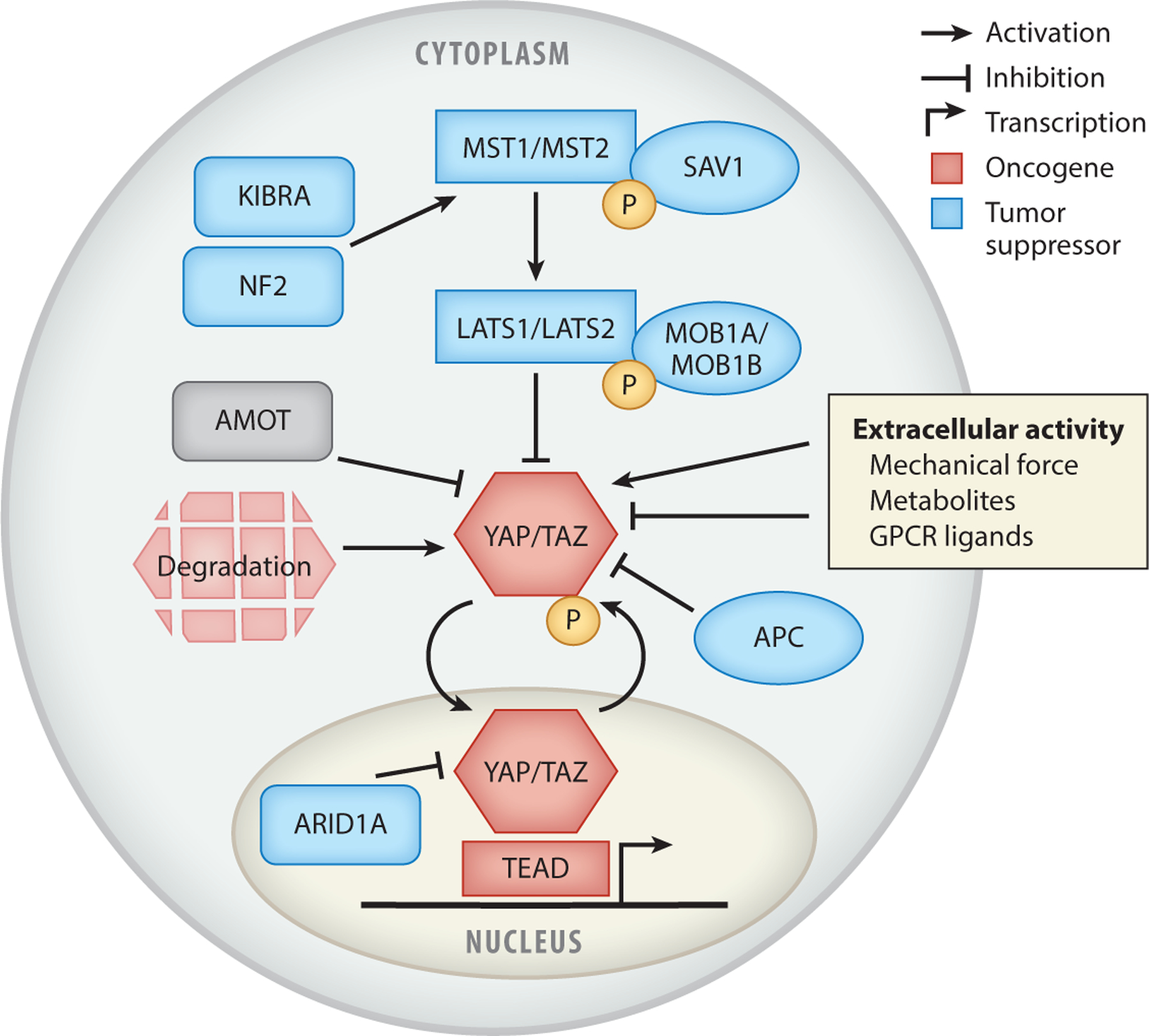
The Hippo pathway in cancer. YAP and TAZ are phosphorylated (represented by P) by the core kinase cascade, enriched in the cytoplasm, and degraded. When not phosphorylated, YAP and TAZ are in the nucleus, where they activate transcription. YAP and TAZ are strongly influenced by extracellular activity, which can promote or inhibit their activity. Tumor suppressors that are frequently mutated in cancer to lead to the activation of YAP and TAZ are highlighted in blue. Oncogenes that frequently exhibit copy number gains in cancer are in red. The component in gray is not frequently mutated in cancer but is discussed in this review.

The core components of the Hippo pathway were initially discovered as a series of highly conserved tumor suppressors in *Drosophila* that cause massive overgrowth in homozygous mutant clones. These tumor suppressors were biochemically linked in a kinase cascade impinging on gene transcription (Pan 2022). In mammals, this pathway comprises the most upstream kinase MST1/MST2 (named *hippo* in *Drosophila*), which acts with its regulatory partner SAV1 to phosphorylate and activate LATS1/LATS2 and its regulatory partner MOB1A/MOB1B, which in turn phosphorylate and inactive the transcriptional coactivators YAP and TAZ (Ma et al. 2019, Zheng & Pan 2019) (Figure 2). When phosphorylated at their canonical Hippo phosphorylation sites, YAP and TAZ are enriched in the cytoplasm by the 14–3-3 complex of proteins and are subsequently degraded (Dong et al. 2007; Lei et al. 2008; Liu et al. 2010; Zhao et al. 2007, 2010). When Hippo signaling is low, YAP and TAZ, located in the nucleus, bind to the TEF/TEAD family of transcription factors, composed of TEAD1, TEAD2, TEAD3, and TEAD4, to activate target gene expression (Neto-Silva et al. 2010, Zanconato et al. 2015, Zhao et al. 2008). Thus, subcellular localization of YAP and TAZ, though imperfect, is often used as a readout of Hippo signaling and YAP/TAZ activity (Chen et al. 2015). Additional proteins mediate the regulation of the Hippo pathway, such as neurofibromin-2 (NF2), a classic tumor suppressor that underlies the autosomal-dominant disorder neurofibromatosis type II. NF2 promotes Hippo signaling by spatially organizing the MST1/MST2–LATS1/LATS2 kinase cascade at the plasma membrane, with loss of NF2 causing hyperactivated YAP/TAZ, a major mechanism by which YAP/TAZ becomes activated in cancer (Hamaratoglu et al. 2006, Yin et al. 2013, Zhang et al. 2010).

By mediating the transcriptional output of the Hippo pathway, YAP and TAZ are potent regulators of cell proliferation, survival, stem cell characteristics, development, and organ size (Ma et al. 2019, Piccolo et al. 2014, Zheng & Pan 2019) and are potent oncogenes (Zanconato et al. 2016). In The Cancer Genome Atlas, mutations in Hippo pathway components occur in approximately 10% of cancers, with the Hippo pathway listed as one of the 10 canonical signaling pathways most frequently altered in cancer, though mutations in YAP and TAZ themselves are rare (Sanchez-Vega et al. 2018). YAP/TAZ activation is sufficient to lead to cancers in mice, such as hepatocellular carcinoma and cholangiocarcinoma (Dong et al. 2007, Yimlamai et al. 2014, Zhou et al. 2009), rhabdomyosarcoma (Tremblay et al. 2014), uveal melanoma (Li et al. 2019), and malignant peripheral nerve sheath tumors (Wu et al. 2018). Aberrant YAP/TAZ activity drives cancer stem cell traits (Castellan et al. 2021, Cordenonsi et al. 2011), immune evasion by regulating PD-L1 ( Janse van Rensburg et al. 2018), resistance to cancer therapies (Kapoor et al. 2014, Lin et al. 2015), and other downstream oncogenic signaling pathways such as Myc (Cai et al. 2018, Ziosi et al. 2010) and Notch (Yimlamai et al. 2014).

## YAP- AND TAZ-FUSION-ASSOCIATED TUMORS

**Figure 3:**
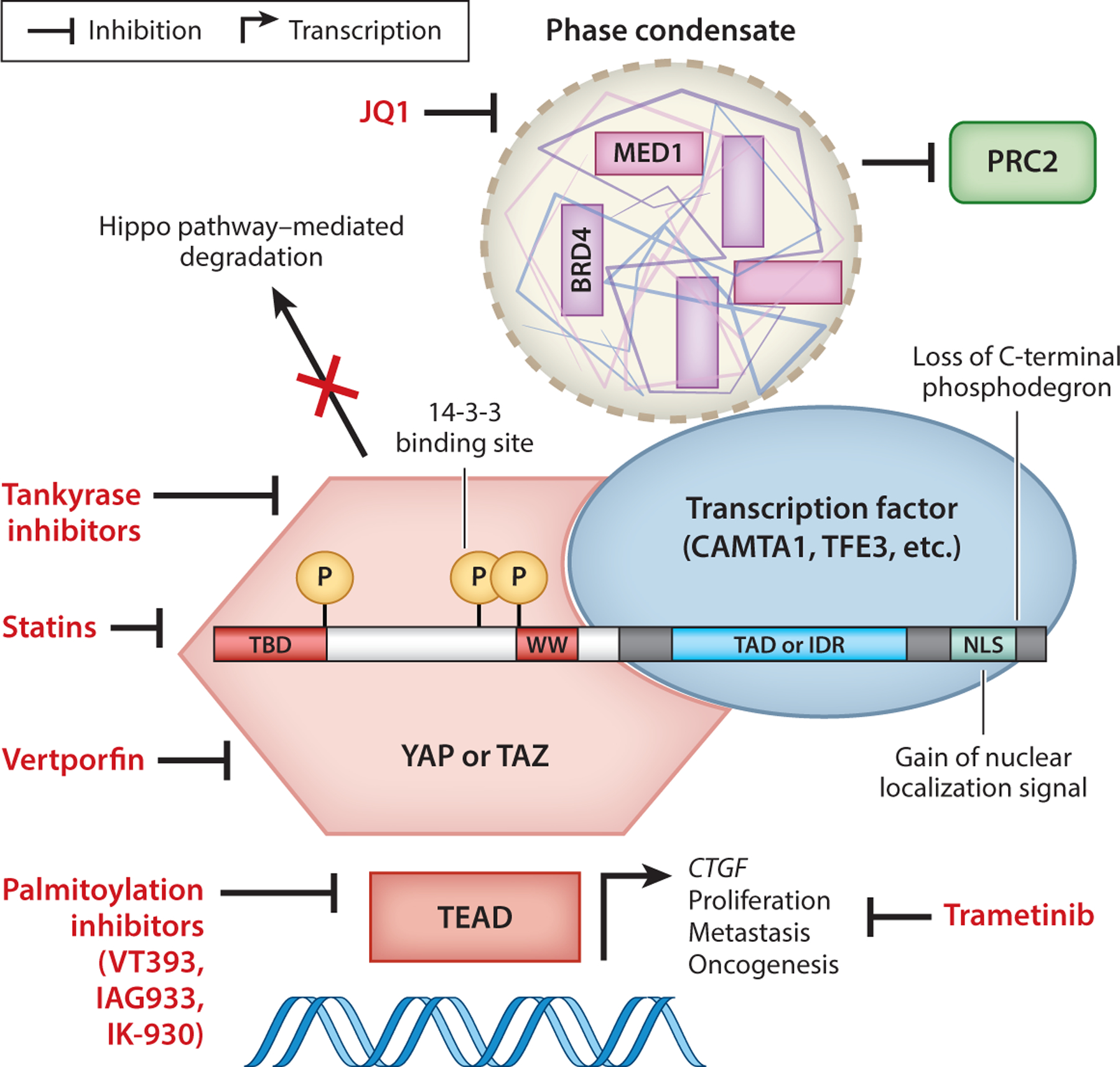
The structure and function of YAP/TAZ fusion proteins. YAP/TAZ fusion proteins are localized to the nucleus, are resistant to Hippo-driven degradation, and drive an oncogenic program. The canonical C-terminal fusion partner may participate in phase condensates to hyperactivate transcription and inhibit Polycomb-dependent gene repression (PRC2). Proposed therapeutic inhibitors of YAP/TAZ fusion oncoproteins are shown in red. Abbreviations: IDR, intrinsically disordered region; NLS, nuclear localization signal; TAD, transcription activation domain; TBD, TEAD-binding domain.

We discuss a select group of tumors in which *YAP1* and *WWTR1* gene fusions (producing YAP and TAZ fusion proteins) are recurrent and distinctive. We list a broader spectrum of reported likely oncogenic YAP and TAZ fusion genes in tumors and their respective anatomical locations in Figure 1, and their relevant gene breakpoints are given in Supplemental Table 1. The structure of YAP and TAZ and the typical YAP and TAZ fusion proteins are depicted in Figure 3 and Supplemental Figure 1.

### Epithelioid Hemangioendothelioma

EHE is a vascular sarcoma with often unpredictable behavior that can occur in individuals of any age at any primary site, most commonly in the liver followed by lungs and bone/soft tissue (Sardaro et al. 2014, Stacchiotti et al. 2021). Most cases (~90%) exhibit a *WWTR1::CAMTA1* gene fusion, which is unique among vascular tumors (Errani et al. 2011, Tanas et al. 2011). Half of EHE tumors exhibit no secondary genetic alterations, although more aggressive tumors may exhibit additional *CDKN2A/CDKN2B* loss (Seavey et al. 2022, Seligson et al. 2019). The small minority of patients lacking *WWTR1::CAMTA1* gene fusions show distinct morphology and harbor instead a *YAP1::TFE3* gene fusion, which may be associated with an indolent disease course (Antonescu et al. 2013, Dermawan et al. 2021a, Rosenbaum et al. 2020).

EHE with a *WWTR1::CAMTA1* gene fusion is characterized by cords or nests of epithelioid cells in a myxohyaline stroma, often containing intracytoplasmic vacuoles (Sardaro et al. 2014, Stacchiotti et al. 2021) (Figure 1a). EHE with a *YAP1::TFE3* gene fusion, on the other hand, commonly harbors epithelioid cells with voluminous cytoplasm arranged in large nests (Antonescu et al. 2013, Dermawan et al. 2021a) (Figure 1d). Both CAMTA1 and TFE3 have been established as useful markers by immunohistochemistry for the diagnosis of EHE (Anderson et al. 2021, Antonescu et al. 2013, Dermawan et al. 2021a, Doyle et al. 2016, Flucke et al. 2014, Shibuya et al. 2015). The function of these two transcription factors remains unknown, but CAMTA1 is expressed mostly in the brain and causes ataxia when knocked out in mice (Long et al. 2014, Tanas et al. 2011) and TFE3 rearrangements are frequently the hallmark of Xp11 translocation renal cell carcinoma (Argani et al. 2001, 2002, 2003) and alveolar soft part sarcoma (Ladanyi et al. 2001).

The *WWTR1::CAMTA1* gene fusion consists of the first two or three exons of *WWTR1* fused in-frame to exons 8 or 9 of *CAMTA1* (Errani et al. 2011, Patel et al. 2015, Tanas et al. 2011). The resulting fusion protein thus retains the N terminus of TAZ—consisting of its TEAD-binding domain, WW domain, and three Hippo phosphorylation sites (including the 14–3-3 binding site)—fused to the bulk of CAMTA1 that includes a transcription activation domain (TAD) and a nuclear localization signal (NLS) (Tanas et al. 2016) (Figure 3; Supplemental Figure 1). The *YAP1::TFE3* gene fusion instead contains exon 1 of *YAP1* fused in-frame to exon 4 of *TFE3*, with the resulting fusion protein consisting of the TEAD-binding domain and a single Hippo phosphorylation site of YAP fused to the TAD and the basic helix-loop-helix leucine zipper domain of TFE3 (Antonescu et al. 2013). In contrast to TAZ in the TAZ::CAMTA1 fusion protein, YAP in the YAP::TFE3 fusion protein has lost its two WW domains and the critical S127 Hippo phosphorylation site that binds to 14–3-3 (Zheng & Pan 2019).

Recently, a case series demonstrated alternate C-terminal fusion partners to TAZ in cardiac EHE, with *WWTR1* fused in-frame to *MAML2* or to *ACTL6A* (Suurmeijer et al. 2020) (Figure 1b,c). MAML2 is a transcriptional coactivator for Notch signaling and is frequently rearranged in mucoepidermoid carcinoma (Sekine et al. 2019; Tonon et al. 2003; Wu et al. 2002, 2005). ACTL6A is a subunit of the SWI/SNF complex and has been suggested to enhance the activity of YAP/TAZ ( Ji et al. 2018, Suurmeijer et al. 2020).

### Supratentorial Ependymoma

Ependymomas are rare central nervous system tumors that arise in the posterior fossa, supratentorium, or spinal cord (Saleh et al. 2022). Two genetically defined subsets of supratentorial ependymomas have been recently described. One is characterized by *YAP1::MAMLD1* gene fusions or rarer *YAP1::FAM118B* gene fusions, and the other is defined by zinc finger translocation-associated (ZFTA) (C11orf95) fusions (Pajtler et al. 2015, Parker et al. 2014). The YAP fusion subset of tumors had a higher grade (grade II or III), a propensity to form in younger patients, a better prognosis, and a lower mutational burden than the ZFTA subset of ependymomas (Pajtler et al. 2015). The resulting YAP fusions retain the TEAD-binding domain, its WW domain, and four of five Hippo phosphorylation sites while notably missing the most C-terminal Hippo phosphorylation site (S397) previously shown to be critical for Hippo-dependent proteasomal degradation (Pajtler et al. 2015, Zhao et al. 2007).

While all the fusion proteins we have discussed so far have YAP or TAZ as the N-terminal fusion partner, the ZFTA fusion subset of supratentorial ependymomas includes rare cases containing *ZFTA::YAP1* gene fusions, in which the entire coding frame of YAP as a C-terminal attachment is fused to the zinc fingers of ZFTA (Kupp et al. 2021, Parker et al. 2014).

### Meningioma

Meningioma is the most common primary central nervous system tumor and is more common in adults, though it can be associated in children with neurofibromatosis type II. Rare pediatric meningiomas have been recently identified to have *YAP1::MAML2*, *YAP1::PYGO1*, or *YAP1::LMO1* gene fusions. Importantly, YAP fusions seemed to arise in pediatric meningiomas without *NF2* mutations (Sievers et al. 2020). A follow-up study also identified *YAP1::FAM118B* gene fusions, the same seen in supratentorial ependymoma, in wild-type *NF2* meningiomas (Schieffer et al. 2021).

### Poroma and Porocarcinoma

Poromas are usually solitary nodular lesions that show terminal sweat gland differentiation and have a malignant counterpart known as porocarcinoma (Sekine et al. 2019). Recent sequencing of these tumors demonstrated that 90% of poromas and ~70% of porocarcinomas exhibit *YAP1* or *WWTR1* gene fusions, with *YAP1* fused to either *MAML2* or *NUTM1* and *WWTR1* fused to *NUTM1*. In all of these fusion proteins, the TEAD-binding domain of YAP and TAZ is retained, with some variability in the presence of the WW domain or Hippo phosphorylation sites, and MAML2 or NUTM1 contributes its TAD (Sekine et al. 2019).

### Additional Tumors with YAP/TAZ Fusions

In addition to meningiomas, poromas, and porocarcinomas, *YAP1::MAML2* gene fusions have been detected in most metaplastic thymomas (Vivero et al. 2020, Zhao et al. 2021), about one-third of retiform hemangioendotheliomas and composite hemangioendotheliomas (Antonescu et al. 2020, Koutlas et al. 2021) (Figure 1e,f), a specific morphologic variant of myxoinflammatory fibroblastic sarcoma (Perret et al. 2022), a series of malignant undifferentiated epithelioid neoplasms (Dermawan et al. 2023), schwannoma (Karajannis et al. 2022), and nasopharyngeal carcinoma (Valouev et al. 2014). Clear cell stromal tumor of lung (CCST-L) (Agaimy et al. 2021, Dehner et al. 2022, Dermawan et al. 2021b), an extremely rare, recently recognized neoplasm, and a recently described cutaneous fibromyxoid neoplasm (Patton et al. 2022) showed *YAP1::TFE3* gene fusions similar to the rare EHE subset. But, unlike in EHE, the breakpoint in CCST-L occurs in exon 4 of *YAP1* (Agaimy et al. 2021, Antonescu et al. 2013, Dehner et al. 2022, Dermawan et al. 2021b). Last, a MUC4-negative variant family of tumor in the spectrum of sclerosing epithelioid fibrosarcoma and low-grade fibromyxoid sarcoma shows characteristic *YAP1::KMT2A* gene fusions (Kao et al. 2020, Puls et al. 2020). KMT2A, an H3K4 histone methyltransferase, is involved in hematological malignancies (Bataller et al. 2021).

Fusion genes containing *WWTR1* have been identified in additional tumors. Cases of epithelioid hemangioma and pseudomyogenic hemangioendothelioma have shown *WWTR1::FOSB* gene fusions. An intra-abdominal spindle cell soft tissue sarcoma in the colon wall with associated endometriosis was found to have a *WWTR1::AFF1* gene fusion (Dashti et al. 2022), and a case of ossifying fibromyxoid tumor showed a *KDM2A::WWTR1* gene fusion, with the entire coding frame of *WWTR1* as a C-terminal attachment fused to *KDM2A* (Kao et al. 2017).

## MODELS OF YAP/TAZ FUSION GENE–DRIVEN TUMORS

YAP/TAZ fusion proteins can define the molecular events present in rare tumors, such as EHE, and subsets of other tumors, such as supratentorial ependymoma. Early studies quickly began to show that a large spectrum of these gene fusions is pathogenic (Supplemental Table 2), ushering in a new era in which targeting these gene fusions may be therapeutic for a variety of cancers.

As the first discovered YAP/TAZ fusion protein, TAZ::CAMTA1 was quickly shown to drive transformation and anchorage-independent growth in 3T3 fibroblast cells, MS1 endothelial cells, and SW872 liposarcoma cells (Driskill et al. 2021, Merritt et al. 2021, Tanas et al. 2016). Furthermore, expression of TAZ::CAMTA1, but not TAZ, CAMTA1, or the truncated TAZ or CAMTA1 present in the fusion protein, is sufficient to induce tumorigenesis. Therefore, TAZ::CAMTA1 has gain-of-function properties that cannot be recapitulated from expression of any single part (Tanas et al. 2016). Sekine et al. (2019), studying poroma and porocarcinoma, later demonstrated a similar phenotype, showing that the fusion proteins YAP::MAML2, YAP::NUTM1, and TAZ::NUTM1 each had the capacity to transform 3T3 cells and immortalized human dermal keratinocytes (Sekine et al. 2019). Of note, the cell lines AM-38 (glioblastoma), ES-2 (ovarian carcinoma), and SAS (head and neck carcinoma) were identified to have *YAP1::MAML2* gene fusions that were required for cell fitness (Picco et al. 2019).

Working in vivo, multiple studies demonstrated that several YAP fusion proteins, such as YAP::FAM118B, YAP::MAML2, YAP::MAMLD1, YAP::SS18, and YAP::TFE3, can induce brain tumors and/or muscle tumors in mice (Hu et al. 2023; Park et al. 2015; Szulzewsky et al. 2020, 2021, 2022; Takadera et al. 2020). Similarly, TAZ::CAMTA1 induced MS1 xenograft growth in nude mice and the formation of metastases to the lung (Driskill et al. 2021). TAZ::CAMTA1 and YAP::TFE3 fusion proteins also drove 3T3 or SW872 xenograft growth in NSG mice (Merritt et al. 2021). Furthermore, mouse neural stem cells that express ZFTA::YAP readily form tumors when implanted into the mouse brain (Parker et al. 2014).

Indeed, because many of these YAP and TAZ fusion proteins are tumorigenic, an open question is whether these fusion proteins were themselves sufficient to result in the formation of their respective tumors. Twin studies using genetically engineered mice showed that the endothelial cell–specific expression of the fusion protein TAZ::CAMTA1 is sufficient to cause EHE (Driskill et al. 2021, Seavey et al. 2021). Seavey et al. (2021) further demonstrated that whole-body ubiquitous expression of TAZ::CAMTA1 resulted in a distribution of EHE similar to that resulting from endothelial cell–specific expression of TAZ::CAMTA1, suggesting that TAZ::CAMTA1 specifically drives EHE (Seavey et al. 2021). Furthermore, in vivo expression of TAZ::CAMTA1 in conjunction with loss of CDKN2A enabled the development of a TAZ::CAMTA1-dependent implantable EHE cell line (Seavey et al. 2023). Using doxycycline-inducible TAZ::CAMTA1 transgenic mice, we showed that turning off expression of TAZ::CAMTA1 could reverse the formation of EHE, demonstrating that the fusion gene is required for continued progression of EHE (Driskill et al. 2021). Thus, TAZ::CAMTA1 appears to be sufficient and required for EHE development and progression, implicating it as an intriguing molecular target for patients with EHE (Seavey et al. 2022). Moreover, expression of the fusion proteins YAP::MAMLD1 and ZFTA::YAP of ependymoma in cortical progenitors led to ependymoma-like tumors in mice (Hu et al. 2023), suggesting many YAP and TAZ fusion proteins are sufficient to result in their respective tumors.

## MECHANISMS OF YAP/TAZ FUSION PROTEINS

To date, a variety of YAP and TAZ fusion proteins have been shown to be oncogenic or even sufficient to result in the tumors in which they were first found. Further studies revealed that these fusion proteins, via a variety of mechanisms, lead to supraphysiological YAP/TAZ activity by grafting new TADs that enhance transcriptional activity and by insulating the fusion proteins from negative regulation by Hippo signaling.

### YAP and TAZ Transcriptional Output

From the structures of the fusion proteins described above, it is clear that the TEAD-binding domain of YAP or TAZ, which mediates their transcriptional output, must be included. Indeed, the fusion proteins TAZ::CAMTA1, TAZ::NUTM1, YAP::FAM118B, YAP::MAML2, YAP::MAMLD1, YAP::NUTM1, YAP::SS18, and YAP::TFE3 all induce canonical YAP/TAZ target signatures and often exhibit genome-wide chromatin association similar to that of YAP and TAZ and TEAD (Merritt et al. 2021, Pajtler et al. 2019, Sekine et al. 2019, Szulzewsky et al. 2020, Tanas et al. 2016). Furthermore, the cell lines ES-2, SAS, and AM-38, all of which harbor the *YAP1::MAML2* gene fusion, show a unique YAP-conserved gene signature (Picco et al. 2019). EHE, whether found in patients or genetically engineered in mice, shows a YAP/TAZ gene signature (Driskill et al. 2021, Seavey et al. 2021).

Multiple studies in turn have demonstrated that YAP/TAZ fusion genes require TEAD to initiate oncogenesis (Figure 2c). Mutation of the S51 site, which is required for TEAD binding, of TAZ in TAZ::CAMTA1 completely abrogates the ability of the fusion oncoprotein to transform 3T3 cells (Tanas et al. 2016). Similarly, mutation of the analogous critical TEAD binding site residue S94 of YAP in YAP::TFE3 also eliminated its ability to transform 3T3 cells (Merritt et al. 2021). Knockdown of TEAD4 and TEAD1 also reduced the ability of TAZ::CAMTA1 and YAP::TFE3 to transform 3T3 cells (Merritt et al. 2021, Tanas et al. 2016). The ES-2 and AM-38 cell lines, both of which harbor *YAP1::MAML2* gene fusions, also showed a cell fitness requirement for TEAD1 in a CRISPR dropout screen (Picco et al. 2019).

In vivo, mutation of the TEAD binding site of YAP in YAP::MAML2, YAP::MAMLD1, YAP::FAM118B, YAP::SS18, and YAP::TFE3 significantly reduced the oncogenic potential of these protein fusions to initiate brain tumors in mice (Pajtler et al. 2019; Szulzewsky et al. 2020, 2022). Similarly, short interfering RNA–mediated knockdown of TEAD1–TEAD4 inhibited the activity of these YAP fusion proteins (Szulzewsky et al. 2020). Expression of a TEAD2 dominant-negative protein also inhibited the ability of TAZ::CAMTA1 to induce the formation of EHE-like vascular tumors and lethality in mice. TEAD2 dominant-negative expression alone in endothelial cells in vivo showed no obvious phenotype, suggesting that targeting the TEAD proteins may be well tolerated (Driskill et al. 2021).

### Enriched Nuclear Localization

Studies to date clearly demonstrate that a major function of YAP and TAZ fusion proteins is to initiate a TEAD-dependent YAP/TAZ gene expression program. Furthermore, the YAP and TAZ fusion proteins behave similarly to activated YAP and TAZ, suggesting that these cancers are quintessential YAP- and TAZ-driven tumors (Lamar et al. 2018, Seavey et al. 2022). Indeed, a key mechanism of action appears to be that YAP and TAZ fusion proteins profoundly dysregulate Hippo signaling. Tanas et al. (2016) demonstrated that CAMTA1 contributes an NLS to TAZ::CAMTA1, which ultimately enriches the fusion protein in the nucleus against active Hippo pathway signaling that normally brings TAZ to the cytoplasm. This NLS is functionally required for TAZ::CAMTA1 to transform 3T3 cells and confers resistance to some modalities of Hippo pathway regulation (Tanas et al. 2016).

More broadly, the fusion proteins YAP::FAM118B, YAP::MAML2, YAP::MAMLD1, YAP::NUTM1, YAP::SS18, and YAP::TFE3 were observed to be enriched in the nucleus compared with YAP (Sekine et al. 2019, Szulzewsky et al. 2020). YAP::MAMLD1 also demonstrated nuclear enrichment in human supratentorial ependymomas, and an NLS from the C-terminal MAMLD1 was shown to be required for YAP::MAMLD1 nuclear localization in mouse brain (Pajtler et al. 2019, Szulzewsky et al. 2020). The proteins FAM118B, KMT2A, LMO1, MAML2, NUTM1, PYGO1, SS18, TFE3, and ZFTA have predicted NLSs that may enhance the activity of their respective YAP and TAZ fusions (Kupp et al. 2021, Szulzewsky et al. 2020). Therefore, the fusion protein partners with YAP and TAZ often contribute NLSs that resist canonical Hippo pathway regulation.

Notably, the critical 14–3-3 binding site S89 of TAZ is always retained in all fusion proteins, whereas the analogous S127 site of YAP may be retained (such as YAP::MAMLD1 in ependymoma) or lost (such as YAP::TFE3 in EHE) (Merritt et al. 2021, Pajtler et al. 2015). Whether the 14–3-3 binding site retained in YAP/TAZ fusion proteins is functionally engaged is debated. Although TAZ::CAMTA1 showed decreased 14–3-3 binding (Pajtler et al. 2019, Tanas et al. 2016), this interaction was clearly detected by unbiased mass spectrometry (Driskill et al. 2021). Mutation of the S89 site did not further increase the transformation activity of TAZ::CAMTA1 in 3T3 cells (Tanas et al. 2016). However, knockout of *LATS1/LATS2* or mutation of the three Hippo phosphorylation sites of TAZ::CAMTA1 significantly increased TAZ::CAMTA1’s nuclear localization and activity, and drugs that drive Hippo pathway activity relocalized the fusion protein to the cytoplasm (Driskill et al. 2021).

One possibility to reconcile these data is that different YAP and TAZ fusions, even when they retain the 14–3-3 binding site, may respond differently to Hippo signaling. For example, overexpression of LATS1, MST1, and MOB1 significantly reduced the activity of YAP::SS18 but not YAP::MAMLD1 or YAP::FAM118B fusion proteins (Pajtler et al. 2019, Szulzewsky et al. 2020). However, LATS1/LATS2 knockdown could increase the activity of YAP::MAMLD1, suggesting that the response to Hippo signaling may be context dependent (Szulzewsky et al. 2020). Future studies, perhaps using in vivo genetic and functional analyses of these fusion proteins, may resolve whether the Hippo pathway can still control the nuclear localization of some of these fusion proteins.

### Loss of the C-Terminal Phosphodegron

Although increased or constitutive nuclear localization is a common feature of the reported YAP and TAZ fusion proteins, constitutive nuclear localization of YAP through mutation of its 14–3-3 binding site or fusion with an NLS is insufficient to induce tumorigenesis (Chen et al. 2015, Szulzewsky et al. 2020). Therefore, additional mechanisms are likely required for the oncogenic activity of the fusion genes. An intriguing feature of almost all YAP and TAZ fusion protein is the loss of the most C-terminal phosphodegron that normally targets phosphorylated YAP/TAZ for proteasomal degradation. Indeed, TAZ::CAMTA1, YAP::FAM118B, YAP::MAMLD1, YAP:: SS18, and YAP::TFE3 fusion proteins showed increased stability over their wild-type YAP or TAZ counterpart in cultured cells (Driskill et al. 2021, Szulzewsky et al. 2020). These findings suggest that the oncogenic activity of YAP and TAZ fusions is due to a combination of nuclear localization and loss of their phosphodegron. In support of this view, YAP with a mutated and defective C-terminal phosphodegron and 14–3-3 binding site, but not YAP fused to an NLS, was sufficient to induce brain tumors in mice (Szulzewsky et al. 2020).

### Phase Separation

Liquid-liquid phase separation (LLPS) is a process in which macromolecules form dense liquid-like droplets to facilitate compartmentalization of subcellular functions (Alberti & Hyman 2021, Banani et al. 2017, Shin & Brangwynne 2017). Though challenging to study within living cells under dynamic conditions, LLPS regulates Hippo signaling, fusion oncoprotein activity, and gene transcription (Boulay et al. 2017, Henninger et al. 2021, Sabari et al. 2018, Wang et al. 2022). A recent fascinating study found that the fusion proteins YAP::MAMLD1 and ZFTA::YAP but not wild-type YAP undergo LLPS in ependymoma that is critical to their oncogenesis (Hu et al. 2023). The C-terminal MAMLD1 contains an intrinsically disordered region (IDR) that drives LLPS in the nucleus to promote the transcriptional activity of YAP. LLPS of YAP::MAMLD1 concentrates TEAD and transcriptional coactivation machinery such as BRD4 and MED1 and inhibits gene-repressive Polycomb complex activity. Supporting this argument, addition of the IDR of MAMLD1 to constitutively nuclear YAP was sufficient to result in ependymoma (Hu et al. 2023). As many YAP and TAZ fusion proteins involve fusion partners with IDRs, this mechanism may underlie the oncogenicity of many YAP and TAZ fusion proteins and represent a new therapeutic opportunity.

### YAP and TAZ Fusion Proteins as Activated YAP and TAZ

Taken together, these fusion genes seem to behave as a gain-of-function YAP and TAZ largely insulated from Hippo-mediated inhibition. Some in vivo experiments seem to support this idea. Mice genetically engineered to express a TAZ^S4A^ or YAP^S5A^ mutant protein, in which all Hippo phosphorylation sites of TAZ or YAP are mutated, show a formation of EHE-like tumors similar to that in mice that express TAZ::CAMTA1 (Driskill et al. 2021; Jung et al. 2021a,b). *NF2*-mutant meningiomas and meningiomas with *YAP1::MAML2* gene fusions share a gene expression profile, and meningioma in mice generated from YAP::MAML2 expression is phenotypically consistent with activated YAP expression (Szulzewsky et al. 2022). Furthermore, either loss of both *LATS1* and *LATS2*, leading to activation of wild-type YAP/TAZ, or expression of YAP::MAMLD1 or ZFTA::YAP is sufficient to result in ependymomas in mice, suggesting that the presence of YAP::MAMLD1 or ZFTA::YAP or the loss of LATS1 and LATS2 activates YAP/TAZ to drive ependymoma (Hu et al. 2023).

However, tumors with different YAP/TAZ fusion proteins may show different morphologies. For instance, EHE in patients with *WWTR1::CAMTA1* gene fusions is distinct histologically from EHE in patients with *YAP1::TFE3* gene fusions and even shows different behavior clinically (Antonescu et al. 2013, Dermawan et al. 2021a, Rosenbaum et al. 2020, Seavey & Rubin 2021). Mouse brain tumors formed from YAP::FAM118B, YAP::MAMLD1, or YAP::SS18 fusion protein expression showed distinct morphology and gene expressions profiles from brain tumors with the YAP::TFE3 fusion protein. Indeed, a chromatin immunoprecipitation and sequencing analysis revealed binding of the YAP::TFE3 fusion protein to both TEAD and TFE3 target genes (Merritt et al. 2021, Szulzewsky et al. 2020). Thus, it is conceivable that some C-terminal transcription partners present in YAP and TAZ fusion genes may contribute to the unique target gene expression program that defines a particular fusion-driven tumor.

## FUTURE DIRECTIONS AND DISCOVERIES OF YAP/TAZ FUSION PROTEINS

The studies so far raise interesting questions about why some fusion proteins seem to be specific to a particular disease, such as TAZ::CAMTA1 to EHE, whereas others are more permissive across a range of tumors, such as YAP::MAML2 to poroma, metaplastic thymoma, and glioblastoma, among others. One possibility is that the C-terminal transcription factors confer transcriptional specificity and drive unique genes that allow the formation of the tumor. For instance, both TAZ::CAMTA1 and YAP::TFE3 fusion proteins activate YAP/TAZ target genes but also have their own unique transcriptional targets (Merritt et al. 2021), although it remains unknown whether any of these unique target genes are required for tumorigenesis. Another possibility is that the C-terminal transcription partners are vulnerable to tissue-specific posttranscriptional regulation, such that the fusion protein is expressed or degraded in specific tissue/cell types. It is also possible that chromosomal configuration may facilitate the generation of certain YAP/TAZ fusions. For example, YAP::MAML2, a fusion protein found across multiple cancer types, results from a local chromosomal inversion (Sekine et al. 2019), which may be more common than fusions resulting from translocations. Future studies are needed to distinguish between these and additional possibilities.

Another question concerns how variant *YAP1* and *WWTR1* gene fusions play a role within tumors of similar origin. In the context of vascular tumors, TAZ::CAMTA1 (Errani et al. 2011, Tanas et al. 2011), TAZ::ACTL6A, and TAZ::MAML2 (Suurmeijer et al. 2020) have all been found in EHEs (Figure 1), but TAZ::FOSB has been found in epithelioid hemangioma (Antonescu et al. 2014, Tsuda et al. 2021) and pseudomyogenic hemangioendothelioma (Panagopoulos et al. 2019). Future studies can unravel whether some fusion proteins within the same tumor are truly interoperable or whether each fusion protein results in different behavior and histology. Another intriguing observation in vascular tumors is that TAZ::MAML2 may be found in EHE (Suurmeijer et al. 2020) but that YAP::MAML2 is associated more with composite or retiform hemangioendotheliomas (Antonescu et al. 2020). One possible explanation for how YAP::MAML2 and TAZ::MAML2 may initiate different hemangioendotheliomas comes from studies demonstrating that YAP and TAZ themselves have overlapping but distinct transcriptional targets and effects (Hagenbeek et al. 2018, Plouffe et al. 2018). Additionally, YAP and TAZ have different expression patterns in endothelial cells; in mice, knockout of *Taz* but not *Yap* in endothelial cells recapitulates loss of *Yap* and *Taz* (Ong et al. 2022), which may contribute to the more aggressive nature of TAZ::CAMTA1-associated EHEs versus YAP::TFE3-associated EHEs and potentially different cells of origin for these tumors. These possibilities add another layer to the question of why TAZ::CAMTA1-associated and YAP::TFE3-associated EHEs, in addition to any effects generated by their different C-terminal transcriptional partners, show distinct behavior and histology (Antonescu et al. 2013, Dermawan et al. 2021a, Rosenbaum et al. 2020).

Studies of the limited number of YAP/TAZ fusions identified so far provide a conceptual framework for interpreting new YAP/TAZ gene fusions as they are discovered from cancer sequencing efforts (Figure 3). All YAP and TAZ fusion proteins characterized to date maintain their TEAD-binding domain, present within exon 1 of *YAP1* and exon 2 of *WWTR1*. They often include in-frame fusion to a C-terminal transcription factor with its TAD/IDR and NLS while losing the C-terminal phosphodegron of YAP/TAZ. These features generate a TEAD-dependent chimeric transcription factor with gain-of-function activity. We propose that any YAP or TAZ fusion protein with these key features may play a potential role in tumor initiation, maintenance, progression, or therapy resistance.

## ADDITIONAL HIPPO-DYSREGULATING FUSION PROTEINS

Thus, YAP/TAZ gene fusions potently dysregulate Hippo signaling and drive YAP/TAZ genes through TEAD to initiate tumorigenesis in a wide variety of tumor types. However, these are not the only gene fusions that involve the Hippo pathway and YAP/TAZ. Gene fusions may dysregulate the Hippo pathway by involving other Hippo pathway components. A *LATS1::PSEN1* gene fusion, which results in loss of function of LATS1 and therefore YAP/TAZ activation, has been detected in a malignant mesothelioma cell line that lacks the second *LATS1* allele (Miyanaga et al. 2015). *NF2* gene fusions have also been reported as a mechanism that results in NF2 loss of function and YAP/TAZ activation in radiation-induced and primary meningiomas (Agnihotri et al. 2017, Khan et al. 2020).

Other than the rare fusions that directly inactivate tumor suppressors of the Hippo pathway, other well-defined gene fusions may also dysregulate Hippo signaling and drive YAP/TAZ activation through indirect mechanisms. For example, the SS18::SSX fusion protein of synovial sarcomas dysregulates the Hippo pathway through an insulin growth factor signaling axis to drive YAP/TAZ activation and YAP-/TAZ-/TEAD-dependent tumor growth (Isfort et al. 2019). A similar axis through the FUS::DDIT3 fusion oncoprotein drives a dependency on YAP/TAZ in myxoid liposarcoma (Berthold et al. 2022, Trautmann et al. 2019). The PAX3::FOXO1 fusion protein of alveolar rhabdomyosarcoma upregulates the gene *RASSF4*, which in turn inhibits the Hippo pathway at MST1/MST2 and drives YAP activation (Crose et al. 2014).

As discussed above, *YAP1::MAMLD1* gene fusions define one subtype of supratentorial ependymoma while *ZFTA* fusions define a more aggressive subtype (Pajtler et al. 2015). A subset of these ZFTA fusion proteins is composed of ZFTA::YAP fusions that still interact with TEAD and have enhanced nuclear localization (Pajtler et al. 2015). The ZFTA fusion proteins, including the most common ZFTA::RELA and ZFTA::YAP fusion proteins, share a common gene signature (Kupp et al. 2021). ZFTA::RELA activates genes with TEAD-binding motifs, suggesting that shared target gene specificity of these different fusion proteins drives supratentorial ependymoma (Arabzade et al. 2021).

## TARGETING FUSION-DYSREGULATED YAP/TAZ

Several different therapeutic modalities have emerged to target YAP/TAZ fusion proteins (Figure 3). As discussed above, multiple studies have demonstrated a dependency of YAP/TAZ fusion proteins, like the activity of YAP/TAZ, on the TEAD family of transcription factors (Driskill et al. 2021, Merritt et al. 2021, Pajtler et al. 2019, Szulzewsky et al. 2020, Tanas et al. 2016). For instance, we showed that genetic inactivation of the TAZ::CAMTA1–TEAD signaling axis by overexpression of a dominant-negative TEAD2 could completely prevent the formation of TAZ::CAMTA1-dependent EHE-like vascular tumors in mice (Driskill et al. 2021). Verteporfin is a small molecule that inhibits the YAP–TEAD interaction, albeit with nonspecific activities, and prevents YAP-induced tumor growth (Liu-Chittenden et al. 2012). YAP::FAM118B, YAP::MAML2, YAP::MAMLD1, YAP::SS18, and YAP::TFE3 fusion proteins showed decreased activity after treatment with verteporfin (Szulzewsky et al. 2020, 2022). YAP::MAML2 also showed decreased activity after treatment with inhibitors that block TEAD autopalmitoylation (Szulzewsky et al. 2022, Tang et al. 2021), and one of these molecules, MGH-CP1, could inhibit TAZ::CAMTA1 activity and EHE cell proliferation (Pobbati et al. 2015, Seavey et al. 2023). Phase I clinical trials of TEAD palmitoylation inhibitors VT393, IAG933, and IK-930 are ongoing, including in patients with *YAP1* and *WWTR1* gene fusions (Schmelzle et al. 2023, Tolcher et al. 2022, Yap et al. 2023).

Another intriguing possibility is to block the interaction partners and downstream signaling pathways activated by YAP/TAZ fusion proteins. A CTGF–Ras–MEK–MAPK signaling axis has been reported to be activated by the TAZ::CAMTA1 fusion protein, forming the basis of a clinical trial with the MEK1/MEK2 inhibitor trametinib in advanced EHE (Ma et al. 2022, Seavey et al. 2023). Merritt et al. (2021) found that TAZ::CAMTA1 and YAP::TFE3 fusion proteins are dependent on the histone acetyltransferase complex ATAC for their transcriptional activity. In a similar analysis, we found that TAZ::CAMTA1-expressing endothelial cells are sensitive to statins, which are known drivers of Hippo pathway regulation and which may be associated with slightly increased survival in patients with EHE who were on statins for other reasons (Driskill et al. 2021, Subramaniam et al. 2020). Hu et al. (2023) found that YAP::MAMLD1 required the transcriptional cofactor BRD4 and that YAP::MAMLD1 activity was sensitive to the BRD4 inhibitor JQ1.

Many proteins negatively regulate YAP/TAZ through their WW domains, such as ARID1A and AMOT (Chang et al. 2018, Zhao et al. 2011). While it has been proposed that tankyrase inhibitors, which stabilize AMOT and decrease YAP/TAZ activity, may be an intriguing therapeutic modality to inhibit YAP/TAZ fusion proteins that still retain their WW domains (Lamar et al. 2018, Wang et al. 2016), the WW domains appear to be dispensable in YAP/TAZ fusions. For example, YAP::MAML2 in meningioma may retain or lose its WW domains in different tumors (Sievers et al. 2020), but mutation of the WW domain in TAZ::CAMTA1 had no effect on its transformation ability (Merritt et al. 2021). Thus, although the WW domains are a critical node of regulatory inputs into endogenous YAP/TAZ, they are less likely to serve as regulatory targets in the oncogenic YAP/TAZ fusion proteins.

## CONCLUSIONS

The study of YAP/TAZ fusion proteins has provided novel insights into the potency of Hippo pathway dysregulation in driving cancers. These fusions are profoundly disengaged from Hippo pathway–dependent inactivation at multiple levels and therefore drive supraphysiological YAP and TAZ activation. A striking feature of YAP and TAZ fusions is that they are often detected in and are pathognomonic for rare tumors, such as EHE. In other rare tumors, such as meningioma, YAP and TAZ fusions are detected in a manner that is exclusive of Hippo pathway mutations, suggesting that these tumors are driven by YAP or TAZ activation regardless of the mode of activation. In contrast, *YAP1* and *WWTR1* gene fusions have so far been undetected in some common tumors with prevalent YAP and TAZ activation, such as hepatocellular carcinoma (Driskill & Pan 2021). These findings suggest that the occurrence of YAP/TAZ fusion events, the stability of the fusion proteins, and the transcriptional capability of the fusion proteins are highly tissue-specific. From a practical perspective, YAP/TAZ fusion tumors represent ideal models for the development of novel therapeutics that more broadly target YAP- and TAZ-activated cancers. Future studies will unravel the full spectrum of YAP and TAZ fusion proteins and their clinical and prognostic significance in human cancer and ultimately provide mechanistic and therapeutic insights that will benefit patients.

## Supplementary Material

1
