# Supplementary material for: YAP, TAZ, and Hippo-Dysregulating Fusion Proteins in Cancer
Source: Annu Rev Cancer Biol. Author manuscript; Available in PMC 2025 Jun 9. (PMC12147517; doi:10.1146/annurev-cancerbio-061223-094639)
Supplement: 1 [file NIHMS2020097-supplement-1.pdf]

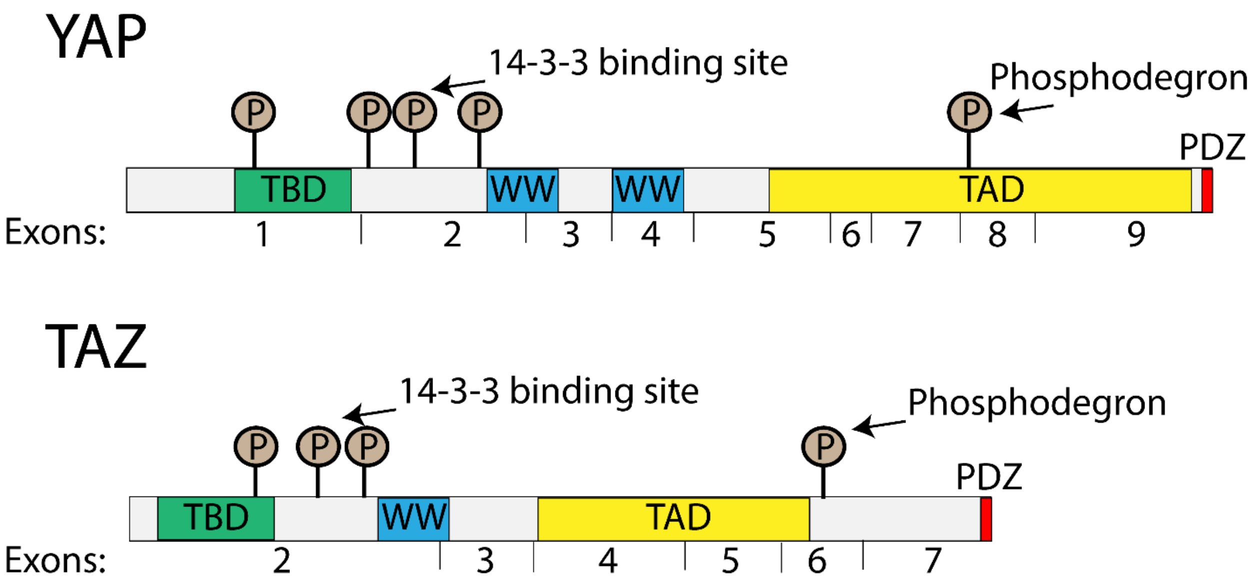


**Supplemental Figure 1.** The structure of YAP & TAZ. Hippo phosphorylation sites are represented by "P". TBD-TEAD binding domain. TAD- transcription activation domain. NLS-nuclear localization signal.

Downloaded from www.annualreviews.org. Guest (guest) IP: 129.112.109.41 On: Thu, 03 Oct 2024 18:30:40

**SUPPLEMENTAL TABLE 1.** Tumors in which YAP/TAZ fusion genes have been reported.

| Tumor | N-terminal protein | C-terminal protein | References |
|---|---|---|---|
| **Nervous System Tumors** | | | |
| Supratentorial ependymoma | YAP (exons 1-5 or 1-6) | MAMLD1 (exons 2-7 or 3-7) | (Pajtler et al 2015, Parker et al 2014) |
| | YAP (exons 1-7) | FAM118B (exons 3-9) | (Pajtler et al 2015) |
| | ZFTA (C11orf95) (exons 1-5) | YAP (exons 1-9) | (Parker et al 2014) |
| Meningioma | YAP (exon 1 or exons 1-5) | MAML2 exons (2-5) | (Sievers et al 2020) |
| | YAP (exons 1-4) | PYGO1 (exons 2-3) | (Sievers et al 2020) |
| | YAP (exons 1-4) | LMO1 (exons 2-3) | (Sievers et al 2020) |
| | YAP (exons 1-7) | FAM118B (exons 3-9) | (Schieffer et al 2021) |
| Glioblastoma | YAP (exons 1-5) | MAML2 (exons 2-5) | (Picco et al 2019) |
| Schwannoma | YAP (exons 1-5) | MAML2 (exons 2-5) | (Karajannis et al 2022) |
| **Head and Neck Tumors** | | | |
| Squamous cell carcinoma of tongue | YAP (exons 1-5) | MAML2 (exons 2-5) | (Picco et al 2019) |
| Nasopharyngeal carcinoma | YAP (exons 1-2) | MAML2 (exons 2-5) | (Valouev et al 2014) |
| **Lung. Breast, & Mediastinum Tumors** | | | |
| Breast carcinoma | YAP (exons 1-7) | STIM1 (exons 2-17) | (Hu et al 2018) |
| Clear cell stromal tumor of lung | YAP (exons 1-4 or 1-5) | TFE3 (exons 7-12) | (Agaimy et al 2021, Dehner et al 2022, Dermawan et al 2021b) |
| Lung adenocarcinoma | YAP (exons 1-3) | C11orf70 (exons 3-4) | (Hu et al 2018) |
| Metaplastic thymoma | YAP (exon 1 or exons 1-5) | MAML2 (exons 2-5) | (Vivero et al 2020) |
| **Gynecological Tumors** | | | |
| Ovarian clear cell carcinoma | YAP (exons 1-5) | MAML2 (exons 2-5) | (Picco et al 2019) |
| Endocervical adenocarcinoma/cervical squamous cell carcinoma | YAP (exon 1) | SS18 (exons 1-11) | (Hu et al 2018) |
| Leiomyoma | TAZ (exons 1-4) | PRKCE (exons 2-31) | (Panagopoulos et al 2022) |
| **Vascular Tumors & Sarcomas** | | | |

Downloaded from www.annualreviews.org. Guest (guest) IP: 129.112.109.41 On: Thu, 03 Oct 2024 18:30:40

| | | | |
|---|---|---|---|
| Epithelioid hemangioma | TAZ (exons 1-3) | FOSB (exons 1-4) | (Antonescu et al 2014, Tsuda et al 2021) |
| Composite hemangioendothelioma | YAP (exons 1-5) | MAML2 (exons 2-5) | (Antonescu et al 2020, Koutlas et al 2021) |
| Epithelioid hemangioendothelioma | TAZ (exons 1-2 or 1-3) | CAMTA1 (exons 8-42) | (Errani et al 2011, Tanas et al 2011) |
| | YAP (exon 1) | TFE3 (exons 4-12 or 6-12) | (Antonescu et al 2013, Dermawan et al 2021a, Rosenbaum et al 2020) |
| | TAZ (exons 1-4) | MAML2 (exons 2-5) | (Suurmeijer et al 2020) |
| | TAZ (exons 1-4) | ACTL6A (exons 2-14) | (Suurmeijer et al 2020) |
| Pseudomyogenic hemangioendothelioma | TAZ (exons 1-4) | FOSB (exons 2-4) | (Panagopoulos et al 2019) |
| Retiform hemangioendothelioma | YAP (exons 1-5) | MAML2 (exons 2-5) | (Antonescu et al 2020) |
| **Skin & Soft Tissue Tumors & Sarcomas** | | | |
| Cutaneous fibromyxoid neoplasm | YAP (exon 1) | TFE3 (exons 4-12) | (Patton et al 2022) |
| Intra-abdominal soft tissue sarcoma | TAZ (exons 1-2) | AFF1 (exons 10-21) | (Dashti et al 2022) |
| Malignant undifferentiated epithelioid neoplasm | YAP (exon 1 or 5) | MAML2 (exon 2) | (Dermawan et al 2023) |
| MUC4-negative low grade fibromyxoid sarcoma/Sclerosing epithelioid fibrosarcoma | YAP (exons 1-4 or 1-5) | KMT2A (exons 4-37 or 5-37) | (Kao et al 2020, Puls et al 2020) |
| Myxoinflammatory fibroblastic sarcoma | YAP (exon 1 or exons 1-5) | MAML2 (exons 2-5) | (Perret et al 2022) |
| Ossifying fibromyxoid tumor | KDM2A (exons 1-14) | TAZ (exons 2-16, entire coding frame) | (Kao et al 2017) |
| Poromas & Porocarcinomas | YAP (exon 1 or exons 1-5 or exons 1-6) | MAML2 (exons 2-5) | (Sekine et al 2019) |
| | YAP (exons 1-4 or 1-5) | NUTM1 (exons 2-8 or 4-8) | (Sekine et al 2019) |
| | TAZ (exons 1-3) | NUTM1 (exons 2-8) | (Sekine et al 2019) |

Downloaded from www.annualreviews.org. Guest (guest) IP: 129.112.109.41 On: Thu, 03 Oct 2024 18:30:40

**SUPPLEMENTAL TABLE 2.** Models of YAP/TAZ fusion gene-driven tumors. GEMM- Genetically engineered mouse model

| Fusion Protein | Model | Phenotype | References |
|---|---|---|---|
| TAZ::CAMTA1 | 3T3 (mouse fibroblast cell) expression | Anchorage-independent growth, xenograft growth | (Merritt et al 2021, Tanas et al 2016) |
| | MS1 (mouse endothelial cell) expression | Anchorage-independent growth, xenograft growth with lung metastases | (Driskill et al 2021) |
| | SW872 (liposarcoma cells) expression | Anchorage-independent growth, xenograft growth | (Merritt et al 2021) |
| | GEMM flip-excision fusion expression, endothelial cell-specific expression or ubiquitous expression | Formation of epithelioid hemangioendotheliomas on peritoneum, liver, lungs, soft tissue | (Seavey et al 2021) |
| | GEMM, flip-excision fusion expression with *CDKN2A* loss | More aggressive EHE tumor formation, generation of EHE cell line | (Seavey et al 2023) |
| | GEMM, Tet-responsive fusion expression, endothelial cell-specific expression | Formation of EHE-like tumors of the lung, continued expression required for progression | (Driskill et al 2021) |
| | Patient-derived xenograft line | Resembles EHE, sensitive to sirolimus, transplantable | (Stacchiotti et al 2023) |
| TAZ::NUTM1 | 3T3 cell expression | Anchorage-independent growth | (Sekine et al 2019) |
| | HDK (human dermal keratinocyte cell) expression | Anchorage-independent growth | (Sekine et al 2019) |
| YAP::FAM118B | Somatic cell gene transfer, GFAP or Nestin-dependent expression in brain & muscle | Tumor formation, lethality | (Szulzewsky et al 2020, Takadera et al 2020) |
| YAP::MAML2 | 3T3 cell expression | Anchorage-independent growth | (Sekine et al 2019) |
| | HDK expression | Anchorage-independent growth | (Sekine et al 2019) |
| | AM-38 knockdown | Decreased cell fitness | (Picco et al 2019) |
| | ES-2 knockdown | Decreased cell fitness | (Picco et al 2019) |
| | SAS knockdown | Decreased cell fitness | (Picco et al 2019) |

Downloaded from www.annualreviews.org. Guest (guest) IP: 129.112.109.41 On: Thu, 03 Oct 2024 18:30:40

| | | | |
|---|---|---|---|
| | Somatic cell gene transfer, Nestin-dependent expression | Meningioma formation | (Szulzewsky et al 2022) |
| YAP::MAMLD1 | In utero electroporation of fusion into lateral ventricle or cortical progenitors | Tumor formation that mimics ependymoma, lethality | (Hu et al 2023, Pajtler et al 2019) |
| | Somatic cell gene transfer, GFAP or Nestin-dependent expression in brain & muscle | Tumor formation, lethality | (Szulzewsky et al 2020, Takadera et al 2020) |
| | Neural stem cell expression | Neurosphere formation, xenograft growth | (Hu et al 2023) |
| YAP::NUTM1 | 3T3 cell expression | Anchorage-independent growth | (Sekine et al 2019) |
| | HDK expression | Anchorage-independent growth | (Sekine et al 2019) |
| YAP;:SS18 | Somatic cell gene transfer, GFAP or Nestin-dependent expression in brain & muscle | Tumor formation, lethality | (Szulzewsky et al 2020) |
| YAP::TFE3 | 3T3 expression | Anchorage-independent growth, xenograft growth | (Merritt et al 2021) |
| | SW872 expression | Anchorage-independent growth, xenograft growth, metastases to lung | (Merritt et al 2021) |
| | Somatic cell gene transfer, GFAP or Nestin-dependent expression in brain & muscle | Tumor formation, lethality | (Szulzewsky et al 2020) |
| ZFTA::YAP | Mouse neural stem cells transduced with fusion gene and implanted into mouse brain | Tumor formation, lethality | (Parker et al 2014) |
| | In utero electroporation of fusion into cortical progenitors | Ependymoma formation, lethality | (Hu et al 2023) |

Downloaded from www.annualreviews.org. Guest (guest) IP: 129.112.109.41 On: Thu, 03 Oct 2024 18:30:40

**REFERENCES**

Agaimy A, Stoehr R, Michal M, Christopoulos P, Winter H, et al. 2021. Recurrent YAP1-TFE3 Gene Fusions in Clear Cell Stromal Tumor of the Lung. *Am J Surg Pathol* 45: 1541-49

Antonescu CR, Chen HW, Zhang L, Sung YS, Panicek D, et al. 2014. ZFP36-FOSB fusion defines a subset of epithelioid hemangioma with atypical features. *Genes Chromosomes Cancer* 53: 951-9

Antonescu CR, Dickson BC, Sung YS, Zhang L, Suurmeijer AJH, et al. 2020. Recurrent YAP1 and MAML2 Gene Rearrangements in Retiform and Composite Hemangioendothelioma. *Am J Surg Pathol* 44: 1677-84

Antonescu CR, Le Loarer F, Mosquera JM, Sboner A, Zhang L, et al. 2013. Novel YAP1-TFE3 fusion defines a distinct subset of epithelioid hemangioendothelioma. *Genes Chromosomes Cancer* 52: 775-84

Dashti NK, Dermawan JK, Schoolmeester JK, Halling KC, Antonescu CR. 2022. A novel WWTR1::AFF2 fusion in an intra-abdominal soft tissue sarcoma with associated endometriosis. *Genes Chromosomes Cancer* 61: 497-502

Dehner CA, Sadegh D, Boulos F, Messias N, Wang WL, et al. 2022. Clear cell stromal tumour of the lung with YAP1::TFE3 fusion: four cases including a case with highly aggressive clinical course. *Histopathology* 81: 239-45

Dermawan JK, Azzato EM, Billings SD, Fritchie KJ, Aubert S, et al. 2021a. YAP1-TFE3-fused hemangioendothelioma: a multi-institutional clinicopathologic study of 24 genetically-confirmed cases. *Mod Pathol* 34: 2211-21

Downloaded from www.annualreviews.org. Guest (guest) IP: 129.112.109.41 On: Thu, 03 Oct 2024 18:30:40

Dermawan JK, Azzato EM, McKenney JK, Liegl-Atzwanger B, Rubin BP. 2021b. YAP1-TFE3 gene fusion variant in clear cell stromal tumour of lung: report of two cases in support of a distinct entity. *Histopathology* 79: 940-46

Dermawan JK, DiNapoli SE, Sukhadia P, Mullaney KA, Gladdy R, et al. 2023. Malignant undifferentiated epithelioid neoplasms with MAML2 rearrangements: A clinicopathologic study of seven cases demonstrating a heterogenous entity. *Genes Chromosomes Cancer* 62: 191-201

Driskill JH, Zheng Y, Wu BK, Wang L, Cai J, et al. 2021. WWTR1(TAZ)-CAMTA1 reprograms endothelial cells to drive epithelioid hemangioendothelioma. *Genes Dev* 35: 495-511

Errani C, Zhang L, Sung YS, Hajdu M, Singer S, et al. 2011. A novel WWTR1-CAMTA1 gene fusion is a consistent abnormality in epithelioid hemangioendothelioma of different anatomic sites. *Genes Chromosomes Cancer* 50: 644-53

Hu X, Wang Q, Tang M, Barthel F, Amin S, et al. 2018. TumorFusions: an integrative resource for cancer-associated transcript fusions. *Nucleic Acids Res* 46: D1144-D49

Hu X, Wu X, Berry K, Zhao C, Xin D, et al. 2023. Nuclear condensates of YAP fusion proteins alter transcription to drive ependymoma tumourigenesis. *Nat Cell Biol* 25: 323-36

Kao YC, Lee JC, Zhang L, Sung YS, Swanson D, et al. 2020. Recurrent YAP1 and KMT2A Gene Rearrangements in a Subset of MUC4-negative Sclerosing Epithelioid Fibrosarcoma. *Am J Surg Pathol* 44: 368-77

Kao YC, Sung YS, Zhang L, Chen CL, Huang SC, Antonescu CR. 2017. Expanding the molecular signature of ossifying fibromyxoid tumors with two novel gene fusions: CREBBP-BCORL1 and KDM2A-WWTR1. *Genes Chromosomes Cancer* 56: 42-50

Downloaded from www.annualreviews.org. Guest (guest) IP: 129.112.109.41 On: Thu, 03 Oct 2024 18:30:40

Karajannis MA, Li BK, Souweidane MM, Liechty B, Yao J, et al. 2022. YAP1-MAML2 fusion in a pediatric NF2-wildtype intraparenchymal brainstem schwannoma. *Acta Neuropathol Commun* 10: 117

Koutlas IG, Oetting WS, Burns GM, Gopalakrishnan R, Antonescu CR. 2021. Whole Exome Sequencing Identifies Somatic Variants in an Oral Composite Hemangioendothelioma Characterized by YAP1-MAML2 Fusion. *Head Neck Pathol*

Merritt N, Garcia K, Rajendran D, Lin ZY, Zhang X, et al. 2021. TAZ-CAMTA1 and YAP-TFE3 alter the TAZ/YAP transcriptome by recruiting the ATAC histone acetyltransferase complex. *Elife* 10

Pajtler KW, Wei Y, Okonechnikov K, Silva PBG, Vouri M, et al. 2019. YAP1 subgroup supratentorial ependymoma requires TEAD and nuclear factor I-mediated transcriptional programmes for tumorigenesis. *Nat Commun* 10: 3914

Pajtler KW, Witt H, Sill M, Jones DT, Hovestadt V, et al. 2015. Molecular Classification of Ependymal Tumors across All CNS Compartments, Histopathological Grades, and Age Groups. *Cancer Cell* 27: 728-43

Panagopoulos I, Andersen K, Gorunova L, Davidson B, Micci F, Heim S. 2022. A Novel Cryptic t(2;3)(p21;q25) Translocation Fuses the WWTR1 and PRKCE Genes in Uterine Leiomyoma With 3q- as the Sole Visible Chromosome Abnormality. *Cancer Genomics Proteomics* 19: 636-46

Panagopoulos I, Lobmaier I, Gorunova L, Heim S. 2019. Fusion of the Genes WWTR1 and FOSB in Pseudomyogenic Hemangioendothelioma. *Cancer Genomics Proteomics* 16: 293-98

Downloaded from www.annualreviews.org. Guest (guest) IP: 129.112.109.41 On: Thu, 03 Oct 2024 18:30:40

Parker M, Mohankumar KM, Punchihewa C, Weinlich R, Dalton JD, et al. 2014. C11orf95-RELA fusions drive oncogenic NF-kappaB signalling in ependymoma. *Nature* 506: 451-5

Patton A, Bridge JA, Liebner D, Chung C, Iwenofu OH. 2022. A YAP1::TFE3 cutaneous low-grade fibromyxoid neoplasm: A novel entity! *Genes Chromosomes Cancer* 61: 194-99

Perret R, Tallegas M, Velasco V, Soubeyran I, Coindre JM, et al. 2022. Recurrent YAP1::MAML2 fusions in "nodular necrotizing" variants of myxoinflammatory fibroblastic sarcoma: a comprehensive study of 7 cases. *Mod Pathol*

Picco G, Chen ED, Alonso LG, Behan FM, Goncalves E, et al. 2019. Functional linkage of gene fusions to cancer cell fitness assessed by pharmacological and CRISPR-Cas9 screening. *Nat Commun* 10: 2198

Puls F, Agaimy A, Flucke U, Mentzel T, Sumathi VP, et al. 2020. Recurrent Fusions Between YAP1 and KMT2A in Morphologically Distinct Neoplasms Within the Spectrum of Low-grade Fibromyxoid Sarcoma and Sclerosing Epithelioid Fibrosarcoma. *Am J Surg Pathol* 44: 594-606

Rosenbaum E, Jadeja B, Xu B, Zhang L, Agaram NP, et al. 2020. Prognostic stratification of clinical and molecular epithelioid hemangioendothelioma subsets. *Mod Pathol* 33: 591-602

Schieffer KM, Agarwal V, LaHaye S, Miller KE, Koboldt DC, et al. 2021. YAP1-FAM118B Fusion Defines a Rare Subset of Childhood and Young Adulthood Meningiomas. *Am J Surg Pathol* 45: 329-40

Downloaded from www.annualreviews.org. Guest (guest) IP: 129.112.109.41 On: Thu, 03 Oct 2024 18:30:40

Seavey CN, Hallett A, Li S, Che K, Pobbati AV, et al. 2023. Loss of CDKN2A cooperates with WWTR1(TAZ)-CAMTA1 gene fusion to promote tumor progression in epithelioid hemangioendothelioma. *Clin Cancer Res*

Seavey CN, Pobbati AV, Hallett A, Ma S, Reynolds JP, et al. 2021. WWTR1(TAZ)-CAMTA1 gene fusion is sufficient to dysregulate YAP/TAZ signaling and drive epithelioid hemangioendothelioma tumorigenesis. *Genes Dev* 35: 512-27

Sekine S, Kiyono T, Ryo E, Ogawa R, Wakai S, et al. 2019. Recurrent YAP1-MAML2 and YAP1-NUTM1 fusions in poroma and porocarcinoma. *J Clin Invest* 129: 3827-32

Sievers P, Chiang J, Schrimpf D, Stichel D, Paramasivam N, et al. 2020. YAP1-fusions in pediatric NF2-wildtype meningioma. *Acta Neuropathol* 139: 215-18

Stacchiotti S, Tap W, Leonard H, Zaffaroni N, Baldi GG. 2023. New Molecular Insights, and the Role of Systemic Therapies and Collaboration for Treatment of Epithelioid Hemangioendothelioma (EHE). *Curr Treat Options Oncol*

Suurmeijer AJH, Dickson BC, Swanson D, Sung YS, Zhang L, Antonescu CR. 2020. Variant WWTR1 gene fusions in epithelioid hemangioendothelioma-A genetic subset associated with cardiac involvement. *Genes Chromosomes Cancer* 59: 389-95

Szulzewsky F, Arora S, Arakaki A, Sievers P, Bonnin DAA, et al. 2022. Both YAP1-MAML2 and constitutively active YAP1 drive the formation of tumors that resemble NF2-mutant meningiomas in mice. *Genes Dev*: 2022.05.02.490337

Szulzewsky F, Arora S, Hoellerbauer P, King C, Nathan E, et al. 2020. Comparison of tumor-associated YAP1 fusions identifies a recurrent set of functions critical for oncogenesis. *Genes Dev* 34: 1051-64

Downloaded from www.annualreviews.org. Guest (guest) IP: 129.112.109.41 On: Thu, 03 Oct 2024 18:30:40

Takadera M, Satomi K, Szulzewsky F, Cimino PJ, Holland EC, et al. 2020. Phenotypic characterization with somatic genome editing and gene transfer reveals the diverse oncogenicity of ependymoma fusion genes. *Acta Neuropathol Commun* 8: 203

Tanas MR, Ma S, Jadaan FO, Ng CK, Weigelt B, et al. 2016. Mechanism of action of a WWTR1(TAZ)-CAMTA1 fusion oncoprotein. *Oncogene* 35: 929-38

Tanas MR, Sboner A, Oliveira AM, Erickson-Johnson MR, Hespelt J, et al. 2011. Identification of a disease-defining gene fusion in epithelioid hemangioendothelioma. *Sci Transl Med* 3: 98ra82

Tsuda Y, Suurmeijer AJH, Sung YS, Zhang L, Healey JH, Antonescu CR. 2021. Epithelioid hemangioma of bone harboring FOS and FOSB gene rearrangements: A clinicopathologic and molecular study. *Genes Chromosomes Cancer* 60: 17-25

Valouev A, Weng Z, Sweeney RT, Varma S, Le QT, et al. 2014. Discovery of recurrent structural variants in nasopharyngeal carcinoma. *Genome Res* 24: 300-9

Vivero M, Davineni P, Nardi V, Chan JKC, Sholl LM. 2020. Metaplastic thymoma: a distinctive thymic neoplasm characterized by YAP1-MAML2 gene fusions. *Mod Pathol* 33: 560-65

Downloaded from www.annualreviews.org. Guest (guest) IP: 129.112.109.41 On: Thu, 03 Oct 2024 18:30:40
